# Cement Bypass Dust as an Ecological Binder Substitute in Autoclaved Silica–Lime Products

**DOI:** 10.3390/ma16010316

**Published:** 2022-12-29

**Authors:** Katarzyna Borek, Przemysław Czapik, Ryszard Dachowski

**Affiliations:** Faculty of Civil Engineering and Architecture, Kielce University of Technology, Al. Tysiąclecia Państwa Polskiego 7, 25-314 Kielce, Poland

**Keywords:** CBPD, free CaO, autoclaved products, lime binder replacement, sand–lime bricks

## Abstract

The cement industry is one of the most developed industries in the world. However, it consumes excessive amounts of natural resources and can negatively impact the environment through its by-products: carbon dioxide (CO_2_), cement clinker dust (CKD) and cement bypass dust (CBPD). The amount of dust generated in the cement clinker production process depends largely on the technology used. It typically ranges from 0 to 25% by weight of the clinker, and a single cement plant is capable of producing 1000 tons of CBPD per day. Despite practical applications in many areas, such as soil stabilisation, concrete mix production, chemical processing or ceramic and brick production, the dust is still stored in heaps. This poses an environmental challenge, so new ways of managing it are being sought. Due to the significant content of free lime (>30%) in CBPD, this paper uses cement bypass dust as a binder replacement in autoclaved silica–lime products. Indeed, the basic composition of silicate bricks includes 92% sand, 8% lime and water. The investigation shows that it is possible to completely replace the binder with CBPD dust in the autoclaved products. The obtained results showed that all properties of produced bricks were satisfactory. The study concluded that many benefits could be achieved by using cement bypass dust in the production of bricks, including economic bricks for building, reducing the dependency on natural resources, reducing pollution and reducing negative impacts on the environment.

## 1. Introduction

The most popular materials for building walls in Poland and Europe are autoclaved cellular concrete and sand–lime products [1]. These are products obtained in the process of hydrothermal treatment in autoclaves. They are manufactured using binders (cement, lime and gypsum), aggregates (sand, fly ash and blast furnace slag), water and, in the case of cellular concrete, an additional blowing agent and additives. In silicates, the lime binder is 8% of the mass of the entire mixture. Unfortunately, during the production of quicklime, significant amounts of CO_2_ are generated into the atmosphere, which has a negative impact on the environment, polluting the air and exacerbating the greenhouse effect [2]. In the era of growing demand for construction materials, the use of a lime substitute for waste material could reduce CO_2_ emissions. Such a solution would be in line with the idea of sustainable development in construction [3,4,5].

Most Portland cement is used to make concrete, mortars or stuccos, and competes in the construction sector with concrete substitutes, such as aluminium, asphalt, clay brick, fiberglass, glass, gypsum (plaster), steel, stone and wood. In 2021, the total world production of cement was 4.4 billion tons. In 2021, China cement production was an estimated 2.5 billion tons; in Vietnam, 810 million tons; in India, 330 million tons; and in the US, 92 million tons [6]. According to the Polish Cement Association, cement production in Poland in 2021 reached almost 19.3 million tons, the third highest in Europe [7]. The cement industry is one of the largest emitters of mineral dust, which generates problems with its management and disposal. In Portland cement production, about 90% of emissions are direct emissions from the combustion of fossil fuels, the decomposition of calcium carbonate (mainly limestone) and energy consumption [8,9]. Despite the reuse of significant amounts of raw materials, intermediates or final products, there are some dusts that, due to their chemical composition, cannot be reintroduced into the cement production cycle. These include alkali-, chlorine- and sulphur-rich cement kiln dust (CKD) and cement bypass dust (CBPD) [10]. CBPD can be divided into two types depending on the place from which they are collected: dust collected immediately at the cold end of the furnace, and dust collected after adding limestone powder to prevent clogging of the ducts [11,12]. The quality of the bypass dust depends on the clinker burning technology, the type of raw materials and fuels used and the method for removing dust. It is usually between 0 and 25% of the clinker mass, as reported by researchers [13,14]. These dust types differ in their chemical and mineral composition and physical properties [15].

According to the Polish Cement Association, the amount of dust produced during cement manufacturing is decreasing. Their latest report found that the annual quantity of dusts from cement kilns in Poland was about 1200 tons [16]. Compared to 25,000 tons in Oman, 2.7–3.5 million tons in Egypt, 8 million tons in the UK, and 2.5–12 million tons in the US [13,17,18,19], the amount of 1200 tons appears minor. However, as a single cement plant is capable of producing 1000 tons of CBPD daily [20], the reported dust emission rate is not the same as the total dust quantity generated in Polish cement plants and does not include the CKD and CBPD that are recycled back into the kiln system. The amount of dust so used in Poland is much higher and ranges from 9000 to 25,000 tons a year [21]. In 2016, 15,071 tons of CKD and CBPD were reused in the cement production process [22]. However, as recycled dusts lower the cement quality, research is being conducted into new opportunities for dust management [13].

Because of its high alkali content, CBPD is used to produce binders in which it would act as an activator for components with latent binding properties. Binders formed by mixing CBPD, cement and mineral additives in the form of lime, granulated blast furnace slag or fly ash are used in geotechnical soil stabilisation [23,24,25,26].

The use of CBPD for the production of alkali-activated binders (so-called “geopolymers”), where the high content of water-soluble alkali as well as the presence of active CaO contribute to the binding of aluminosilicates in the presence of alkali-metal silicate, is known in the literature [27,28].

Many researchers have used waste dust as a partial replacement for cement binder in concrete materials [29,30,31]. Al-Harthy et al. [32] investigated the effect of CBPD on mortar–concrete mixtures for the partial replacement of Portland cement in concrete. The results indicated that the overall compressive strength, compared to the control mixture, decreased with increasing content of bypass dust in the tested materials. However, the researchers concluded that a mere 5% exchange of dust to cement does not adversely affect the tested strength parameter.

The overall decrease in compressive strength of concrete with CBPD compared to the control mixture was confirmed by Udoeyo and Hyee [33]. However, based on experimental research, it was noted from the experimental study that a minimal decrease in compressive strength occurred when replacing 20% of Portland cement with CBPD.

Aydin et al. [34], based on their research, concluded that cement kiln dust could be used as a source of CaO in the production of ceramic wall tiles. Mahrous and Yang [35] successfully applied CKD dust to clay bricks. Abdulkareem and Eyada used two types of CKD with sand and cement to produce pressed building brick [36]. Abdel-Gawwad et al. [37] utilized CKD with the waste of red clay bricks and silica fume as the main ingredients to produce unfired building bricks. Based on the above, it can be concluded that the introduction of CKD and CBPD, therefore, has a beneficial effect on the properties of wall materials. In addition, since cement bypass dust consists mainly of calcium oxide (CaO), it becomes highly probable that it can be used as a substitute for lime binder in materials in which lime is one of the basic ingredients.

Lime-based wall materials include sand–lime products (92% quartz sand + 8% lime + water) [4,5] and autoclaved aerated concrete [38]. During hydrothermal treatment in an autoclave, chemical processes take place between the components of individual mixtures, and are responsible for the physical and mechanical properties of the finished products. In sand–lime products, crystalline silica, derived from quartz sand, reacts with lime at an elevated temperature of 180 °C and a pressure 10 bar to form hydrated calcium silicates, such as amorphous C–S–H phase and crystalline tobermorite [39]. The use of a substitute for quicklime in autoclaved products would allow for economic advantages the production process stage, and would additionally have a positive impact on the environment, as demonstrated by Vojvodikova, Prochazka and Bohancova [40,41].

Tkaczewska [42] conducted research on the use of small amounts of CBPD (0.05–5.00% by weight of the binder) with free CaO content <10% for the production of autoclaved materials. Autoclaved mortar samples showed a greater increase in compressive strength. After two days of autoclaving, the strength of mortar containing 5% bypass dust increased to 55.60 MPa, while the strength of Portland cement mortar was only 50.05 MPa. After 28 days of autoclaving, the strength values of these mortars were 64.85 MPa and 69.85 MPa, respectively. The increase in compressive strength of the mortar after autoclaving is attributed to hydrothermal treatment, which allows the formation of the C–S–H phase with a higher degree of crystallization and tobermorite in the form of well-formed needles, allowing the cement mortar to achieve higher strength [42].

The use of waste dust as a substitute for natural lime, a component of the classic sand–lime mix, is also well-known. In patent application EP 3 705 462 A1 [43], the use of dust is limited to a range of 5–60% of one or more quicklime sources. The complete replacement of lime by cement kiln dust in the tests carried out resulted in silicate bricks with a significantly reduced compressive strength compared to products with a typical formulation.

In the literature, it was found that limited amounts of waste dust have been used as cement or lime binder. However, no studies have been carried out to determine conclusively whether lime binder can be completely replaced by CBPD dust. In addition, few data indicate the use of waste dust in lime–silica products. The dusts used to date have contained less than 30% free CaO in their composition [43].

The main objective of this study is, therefore, to see whether the extent to which CBPD can be used in autoclaved products (sand–lime products) can be increased. If this is a possibility, a further question arises as to whether the proportion of bypass dust in the mixture of autoclaved products depends on its phase composition and, in particular, on the free CaO content of the dust. Furthermore, the additional question arises whether those dusts with a high free lime content (>30%) should be treated as lime binders.

## 2. Materials and Methods

### 2.1. Material Characterization

Quartz sand, quicklime and CBPD were used to prepare the mass for autoclaved sand–lime samples. The sand applied in this study was used in the production of silicate bricks at one of the production plants in the Świętokrzyskie Voivodship (Ludynia) in Poland. The lime was sourced from the local Trzuskawica plant (Sitkówka, Poland) and was intended for silica products. The CBPD dust used was taken from a local cement plant. The physical and chemical properties of these raw materials are shown in Table 1.

**Figure 1:**
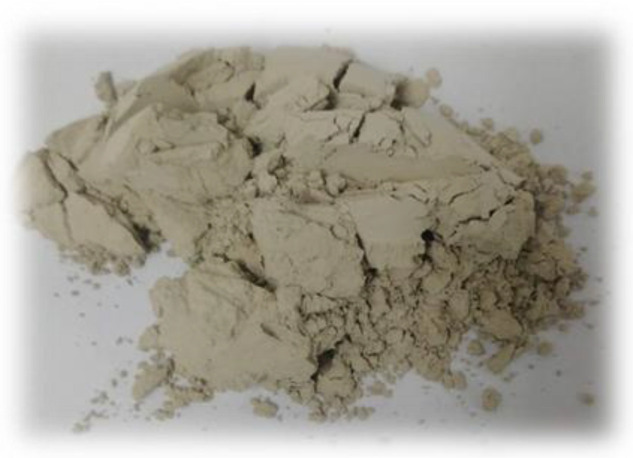
Cement bypass dust (CBPD).

Research indicates that the main component of CBPD (Figure 1) is free lime. Free CaO is highly reactive and reacts rapidly with water. The second largest component is belite, which is the clinker phase formed at the lowest temperature [44]. Chlorides present in the CBPD are condensed in the form of potassium chloride (sylvine). The secondary phases, calcite and quartz, come from unprocessed raw materials.

### 2.2. Preparation of Samples Treated with CBPD

**Figure 2:**
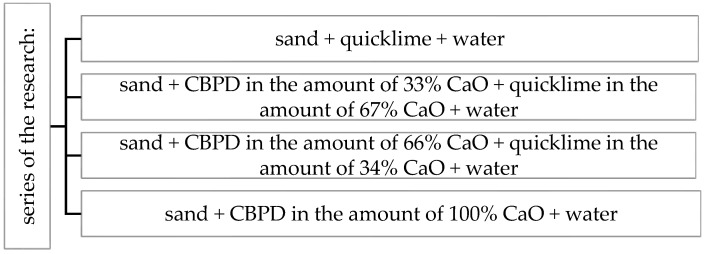
Scheme of the research carried out.

A variable amount of CBPD was introduced into a basic mix consisting of quartz sand (92%), quicklime (8%) and water. Four series of six samples each were performed. The first series included the reference samples and the next three series of samples were modified with CBPD. CaO derived from the quicklime was replaced by free CaO from the bypass dust, dosed at 33, 66 and 100% of the CaO from the lime in the mixture (Figure 2).

**Figure 3:**
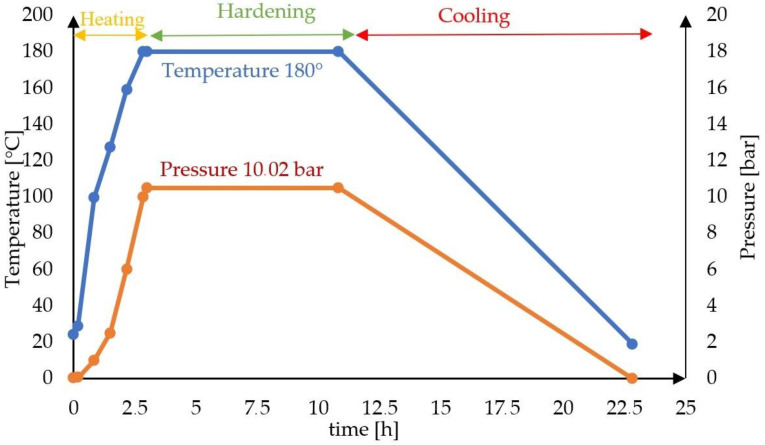
Autoclaving process as a function of time.

Mixtures were prepared with the compositions given in Table 2. After mixing the components and adding water, the mixtures were placed in a sealed glass vessel and dried in an oven at 65 °C for 1 h. Cylindrical samples (with a moisture content of 6–8%) with a diameter and height of 25 mm were then formed by two-stage pressing at pressures of 10 MPa and 20 MPa. The last stage, autoclaving, was divided into three phases shown in Figure 3. In the first stage, the samples were heated in an autoclave to a temperature of 180 °C for 2.5 h. When the assumed temperature and steam pressure were obtained, the second stage began, in which the samples were autoclaved at 180 °C under a saturated steam pressure of 1.002 MPa for 8 h. The third stage lasted 12 h and involved cooling the samples to ambient temperature.

### 2.3. Testing Methods

The paper examines the physical and mechanical properties of samples with the use of cement bypass dust. These tests are necessary to verify the suitability of CBPD dust in sand–lime products used for building walls. The compressive strength was tested 21 days after autoclaving on six cylindrical samples with heights and diameters of 2.5 cm, according to PN-EN 772-1+A1:2015-10 [45]. A Controls 50-C9030 (Controls, Manchester, Barcelona) compression device was used. Prior to testing, the samples were stored in laboratory conditions at room temperature.

The bulk density was determined by the hydrostatic method. Cylindrical samples of autoclaved limestone and sand materials were used for the test, following the scheme below:

1. The limestone and sand samples were completely immersed in water. The water-saturated samples were weighed (to the nearest 0.01 g);
2. One by one, the tested samples were suspended on a thin wire on one of the balance arms and immersed in such a way that the samples and the weighing pan did not come into contact with the vessel, liquid or the table on which the vessel stood;
3. Samples were weighed in water and the measurement results were recorded (to the nearest 0.01 g);
4. The temperature and density of the water in the vessel were determined;
5. The samples were then dried at 103 °C to constant weight;
6. After cooling down, the dry samples were weighed and the measurement result was recorded;
7. The calculations were performed using the Equation (1):

(1) $$\rho =\frac{m_{d}}{V_{0}}\;[\mathrm{kg}/m^{3}]$$

where:

$m_{d}\;$—the weight of the dry sample,
$V_{0\;}$—the volume of the sample including pores calculated according to Equation (2).

(2) $$V_{0}=\frac{m_{1}-m_{2}}{\rho_{w}}$$

where:

$m_{1}$—the weight of the saturated sample, weighed in air, [g]
$m_{2}$—the weight of the saturated sample, weighed in water, [g]
$\rho_{w}$—water density, [g/cm^3^]

In each case, the tests were conducted on six samples of the same batch. The arithmetic mean of the obtained values was assumed as the result of the determination.

Water absorption was determined in accordance with the PN-EN 772-21: 2011 [46] standard. The test was carried out on 6 samples from each batch, using those used for the bulk density analysis. The water absorption was calculated using Equation (3):

(3) $$w=(m_{1}-m_{d})/\;m_{d}\;\times \;100\;[\%]$$

where:

$m_{d}$—weight of the sample after drying,
$m_{1}$—weight of the sample after soaking.

Microstructure and phase composition studies were performed as explanatory tests for the parameters of the physical and mechanical characteristics. Microstructure analysis of silica–lime samples with CBPD was carried out using a Quanta FEG 250 (FEI, Brno, Czech Republic) scanning electron microscope. Measurements were taken in a low vacuum at 5 kV. The test material for the silica–lime products was taken from the damaged samples after the compressive strength test. The tests were, therefore, performed on undusted crumbs of limestone and silica products. To analyse the microstructure of individual samples, magnifications from 1.000 to 20.000× were used and subjected to EDS point analysis.

## 3. Results and Discussion

### 3.1. Compressive Strength

**Figure 4:**
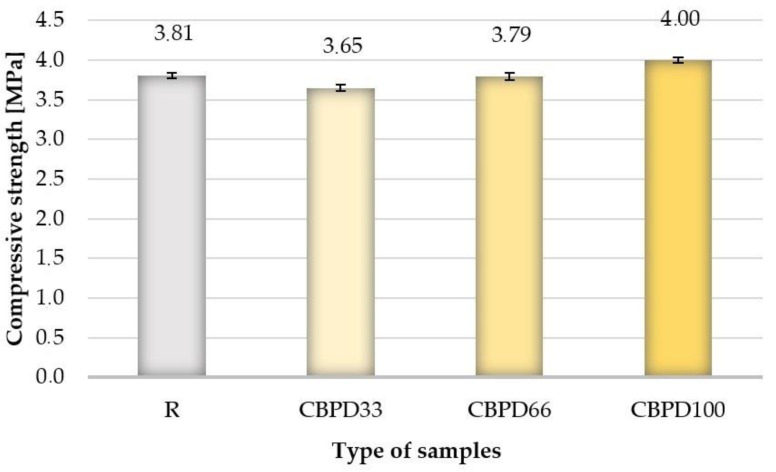
Compressive strength results.

Based on the test results obtained (Figure 4), it can be concluded that modifying the limestone–silica samples with CBPD has a negligible effect on the change in compressive strength. Replacing 100% of free lime in the silica–lime mixture with CBPD dust increases the compressive strength by ~5% (4.00 MPa), compared to the R samples (3.81 MPa). The average compressive strength values of CBPD66 and CBPD33 samples are 0.5% and 4.2% lower, respectively, compared to products with a typical formulation. Temmermans, Tromp and Santamaria noticed a decrease in the examined parameter with an increase in the share of CBPD in autoclaved products [43]. In their paper, they presented that the decrease in compressive strength was more than 50% (28.1 MPa for the reference sample, 12.9 MPa for the sample with 100% dust). In their study, a reduction in the results of the parameter tested was noted for samples with 33 and 66% dust. However, the complete replacement of the lime binder with dust from cement kiln dusting improves the compressive strength. The difference between the compared test results may result from a different share of free CaO in CBPD. In their research, [39] the authors of the patent used dust containing 23.6% of free CaO, which is a lower value compared to the amount of CBPD used in this study.

Tkaczewska [42] used bypass dust as a cement replacement in autoclaved products. Mortar samples with CBPD subjected to hydrothermal treatment showed a greater increase in compressive strength in relation to the samples of typical composition. Tkaczewska explained this phenomenon by the formation of substantial amounts of highly crystallized hydrated calcium silicates, such as tobermorite, resulting from autoclaving.

### 3.2. Bulk Density

**Figure 5:**
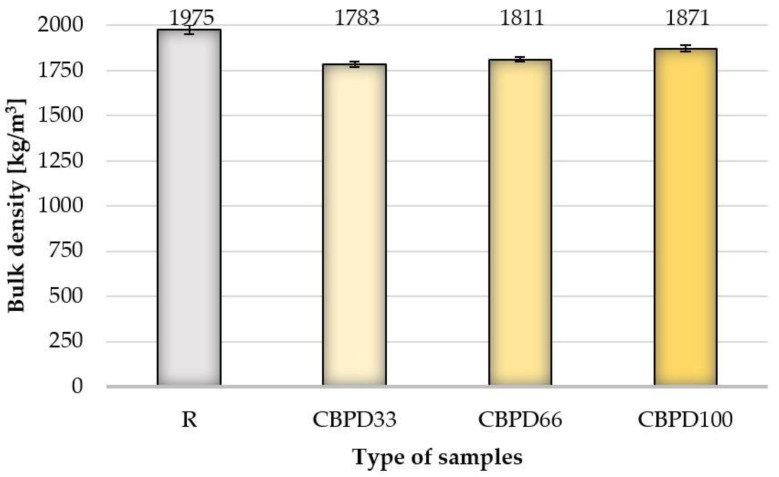
Bulk density results.

Modifying the samples with bypass dust, regardless of its share in the mixture, reduces the volume density in relation to the R samples (Figure 5). The use of dust at 33% relative to the free CaO in the mixture reduces the bulk density to the greatest extent relative to the R samples (about 9.7%). The average bulk density values of the CBPD66 and CBPD100 samples are 8.3% and 5.3% lower in relation to the reference products. The average bulk density of the modified product increases with increasing CBPD content of the sample. A similar phenomenon is illustrated by the specific density of the samples (CBPD33 = 2583 kg/m^3^, CBPD66 = 2604 kg/m^3^, CBPD100 = 2625 kg/m^3^). It is concluded that this may be due to the higher specific density of cement kiln dust, used as a binder replacement, compared to that of quicklime. However, the bulk density of the samples with 100% CBPD is lower compared to the R samples, which may be related to the increase in the open porosity of the modified products.

### 3.3. Water Absorption

**Figure 6:**
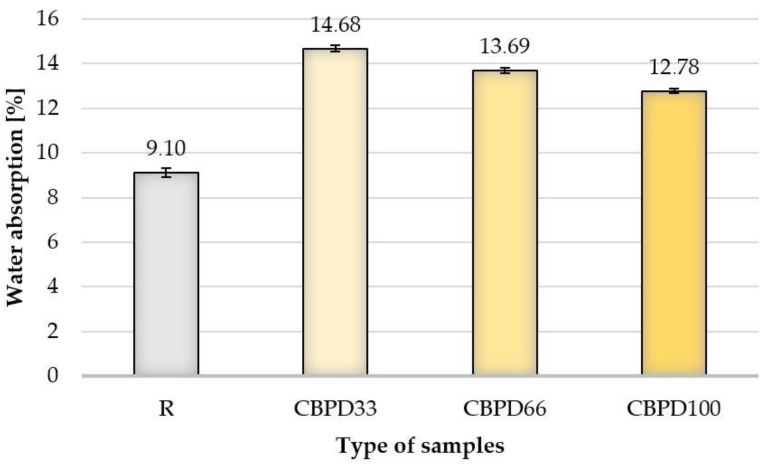
Water absorption results.

Modifying the samples with bypass dust, regardless of its share in the mixture, increases the water absorption in relation to the R samples (Figure 6). The average water absorption values for CBPD100 and CBPD66 samples are 40.4% and 50.4% higher, respectively, compared to the R samples. The use of dust at 33% relative to free CaO in the mixture increases water absorption to the greatest extent in relation to the R samples (by about 61.3%).

The complete replacement of quicklime with dust from the cement kiln dedusting increases the packing density; as a consequence, a denser microstructure is created. The spaces between the aggregate could be sealed as a result of the hydration of the dust grains that previously filled them.

### 3.4. Phase Composition and Microstructure

**Figure 7:**
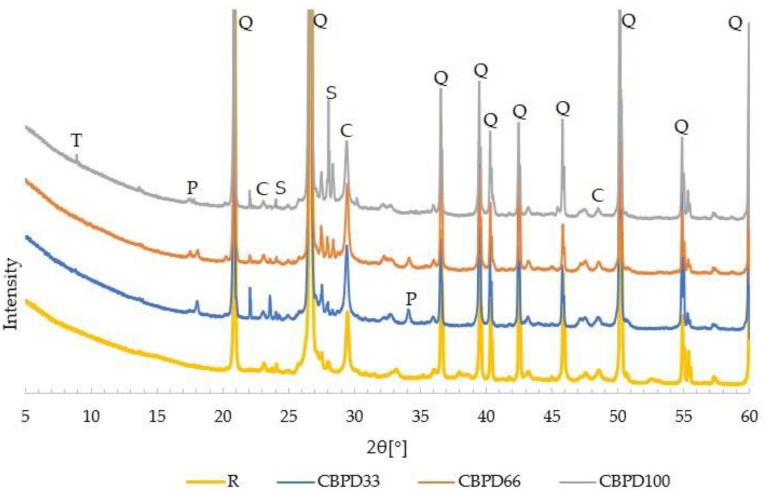
Diffraction pattern of autoclaved sand–lime products with 33%, 66% and 100% CBPD in relation to free CaO, compared to a reference sample. Designations: C—calcite, P—portlandite, S—sylvine, T—tobermorite, Q—quartz.

Diffractograms of silica–lime products are shown in Figure 7. The R sample revealed intense reflections of quartz and calcite. The phase compositions of the products with CBPD and of the R sample are different. All the modified products are dominated by quartz and calcite reflections. The introduction of CBPD resulted in the presence of portlandite and sylvine peaks. As the CBPD content in the products increases, the intensity of quartz (50 2θ°) and portlandite reflections decreases (to show this, a clearer separation of the diffraction patterns would be required). The presence of portlandite indicates that the active lime (contained in free CaO and belite) has neither carbonated nor reacted to form hydrated calcium silicates. Increasing the share of bypass dust increases the intensity of the reflections characteristic of sylvine, particularly visible in the 28 ÷ 30 2θ° angle range. This is justified by the fact that CBPD contains significant amounts of potassium chloride (sylvine) [10]. In the CBPD100 samples, a peak appeared in the 5 ÷ 10 2θ° angle range, characteristic of the crystalline phase, tobermorite. Based on the X-ray analysis, it can be concluded that the formation of hydrated calcium silicates in autoclaved products is possible with the complete replacement of lime with CBPD.

**Figure 8:**
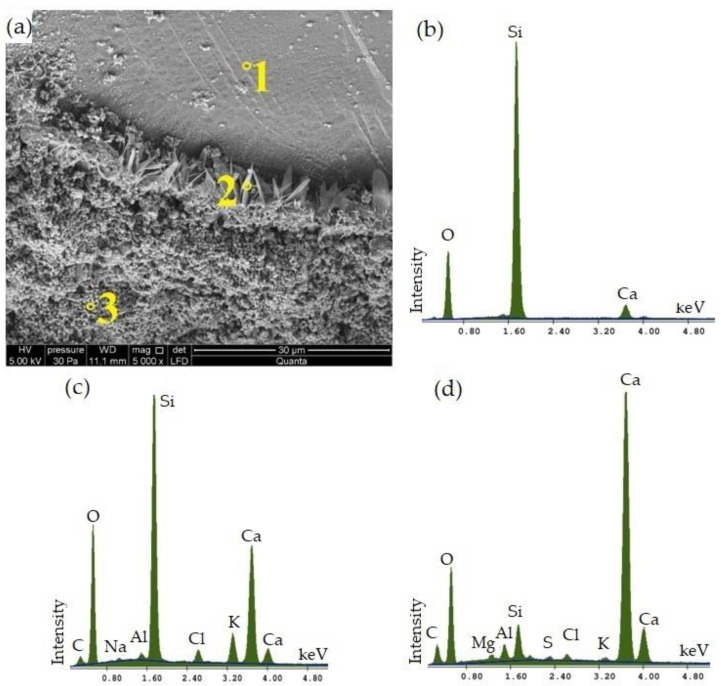
Microscopic SEM image of calcium–silica samples with 33% CBPD at (**a**) 5000× magnification and X-ray microanalysis of its elemental composition at (**b**) 1, (**c**) 2 and (**d**) 3.

The SEM microscopic image of autoclaved silica–lime products with 33% CBPD in the binder mass shown in Figure 8a indicates the formation of phases near the aggregate surface, characteristic of silicate products [47]. In the upper part, a sand grain covered by residual C–S–H phase is visible, as indicated by the result of the EDS analysis (Figure 8b), in which, in addition to an intense quartz signal, a clear calcium signal was obtained. In Point 2, in a small area near the surface of the sand grain, a phase with a higher degree of crystallization—tobermorite [48]—was formed, which is indicated by its lamellar structure and a Ca/Si ratio of 0.8 (Figure 8c). At Point 3, according to EDS analysis (Figure 8d), a spongy C–S–H phase is visible, largely covering the analysed surface of the sample.

**Figure 9:**
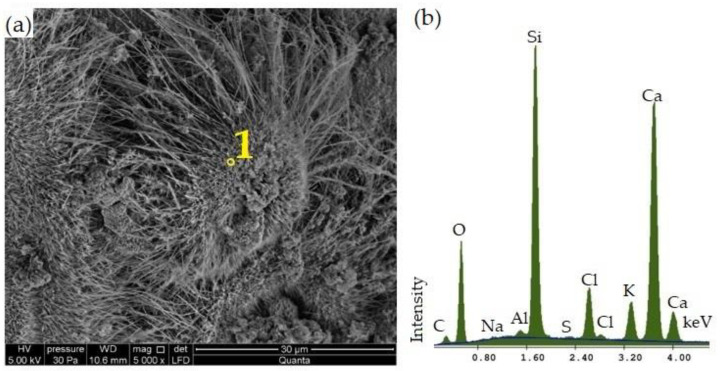
Microscopic SEM image of calcium–silica samples with 100% CBPD at (**a**) 5000× magnification and micro-X-ray analysis of its elemental composition in Point (**b**) 1.

The SEM microscopic image of limestone–silica samples with 100% CBPD seen in Figure 9a shows a different morphology compared to samples modified with a lower dust content. A microstructure created by very densely packed hydrated calcium silicates in the form of well-developed needles was observed over the entire surface of the samples. The short grassy forms develop into long networks of threads filling the areas between the aggregate grains. This can lead to an increase in the tightness of the product, resulting in improved mechanical and physical properties. The formation of hydrated calcium silicates with a higher degree of crystallisation in the structure of the CBPD-modified material is confirmed by the results obtained in the following papers: [42,49]. The EDS analysis (Figure 9b) showed that the resulting structure was characterised by significantly elevated K and Cl readings, which is justified by the proportion of CBPD in the analysed sample.

## 4. Conclusions

The following general conclusions can be drawn from the study:

1. The amount of CBPD dust used in autoclaved products depends on the dust’s chemical composition, primarily on its free CaO content;
2. The use of CBPD dust with >30% CaO content makes it possible to obtain full-value silicate bricks.

The results of this research allow the following specific conclusions to be reached:

1. The complete replacement of quicklime with CBPD contributes to a 5% higher compressive strength with a 5.3% reduction in bulk density. This effect can be considered beneficial from a logistical point of view and the structural properties of the construction material. Lighter construction materials with lower density allow slimmer structures to be erected;
2. Replacing quicklime with CBPD to a lesser extent (33 and 66%) contributes to a greater decrease in density; however, this is accompanied by a reduction in the strength of the sand–lime product;
3. The decrease in the density of sand–lime products due to the addition of CBPD is accompanied by a corresponding increase in water absorption. Thus, it can be concluded that the decrease in density is related to an increase in porosity that allows water to be absorbed;
4. Based on the compressive strength, density, and water absorption tests, it can be concluded that even a small addition of CBPD can significantly affect the properties of the sand–lime product, and this effect can be eliminated by increasing the proportion of dust in the mixture. This may be related to the fact that replacing free CaO from quicklime with that derived from CBPD increases the total amount of binder used. In addition, in the CBPD, not only free lime but also belite shows binding properties, which can positively influence the properties of the material obtained;
5. The modification of the traditional silica–lime mixture with bypass dust alters its phase composition and introduces new phases—portlandite and sylvine—into the system. The increase in modifier content decreases the intensity of the quartz and portlandite reflections and, in the CBPD100 sample, increases the intensity of the peak characteristic of tobermorite;
6. The use of CBPD from cement kilns as a lime substitute does not inhibit the formation of hydrated lime silicates, characteristic of autoclaved products. On the contrary, increasing the proportion of waste dust in the products generates the formation of denser structures with a higher degree of crystallisation, i.e., tobermorite. The key is the content of free CaO in the dust.

The research presented in this article opens up further areas of research related to the elucidation of the microstructural differences and the investigation of practically useful material properties, such as thermal conductivity, water vapour permeability, hygroscopic properties and frost durability. Given the economic and environmental benefits of replacing lime binder in silicate products with waste dust, a study in which the aggregate of products is also replaced should be considered. The substitute for quartz sand could be a waste material containing a high proportion of silica. This would make it possible to produce a product containing only waste materials.

## Figures and Tables

**Table 1:** Physical and chemical properties of the materials used.

**Quartz sand**											
Grain size [mm]			2 sieve = 100%, 0.2 sieve ≥ 97% by weight, 0.09 sieve ≥ 97% by weight,								
Chemical composition [%]			CaO + MgO ≥ 91 MgO ≤ 2.0 CO_2_ ≤ 3.0 SO_3_ ≤ 0.50								
Bulk density [kg/m^3^]			790								
**Ground quicklime**											
Reactivity			60 °C ≤ 2.0 min								
Grain size [mm]			2.5–0.5 = 19% 0.5–0.05 = 81%								
Chemical composition			Si and O, Al, Mg, Fe, K and C								
Density [kg/m^3^]			2650								
**Cement bypass dust (CBPD)**											
Grain size [μm]			od 0.20–15 μm								
Chemical composition [%]	SiO_2_	Al_2_O	Fe_2_O_3_	CaO	MgO	Na_2_O	K_2_O	Na_2_O_e_	Cl	SO_3_	LOI
	15.44	3.42	1.77	52.17	1.31	0.26	6.03	4.22	3.53	1.65	14.40
Phase composition [%]	Free lime	Sylvine		C_2_S (belite)		Calcite	Quartz		Arcanite	Portlandite	
	38.1	16.2		36.9		4.0	2.5		0.9	1.5	
Density [kg/m^3^]			3010								
Specific surface area [cm^2^/g]			5480								

**Table 2:** Composition of mixtures with CBPD (at the moisture content of the mixture of 6–8%).

No.	Sample	Proportion of Quartz Sand		Proportion of CBPD		Proportion of Quicklime	
		[%]	[g]	[%]	[g]	[%]	[g]
1	**R**	92.00	230.00	0.00	0.00	8.00	18.40
2	**CBPD33**	90.04	225.11	4.68	11.69	5.28	13.20
3	**CBPD66**	88.04	220.11	9.28	23.19	2.68	6.70
4	**CBPD100**	85.89	214.73	14.11	35.27	0.00	0.00

## Data Availability

Not applicable.

## References

[1] Statistics Poland (2020). Statistical Analyses. Market of Construction Products Manufactures in 2015–2018.

[2] The Science in Poland. https://naukawpolsce.pl/aktualnosci.

[3] Ajith S., Arumugaprabu V., Szóstak M. (2022). A framework for systematic assessment of human error in construction sites—A sustainable approach. Civil Eng. Arch..

[4] Borek K., Czapik P. (2022). Utilization of waste glass in autoclaved silica–lime materials. Materials.

[5] Borek K., Czapik P., Dachowski R. (2020). Recycled glass as a substitute for quartz sand in silicate products. Materials.

[6] Hatfield A.K.U.S. (2022). Geological Survey, Mineral Commodity Summaries. https://pubs.usgs.gov/periodicals/mcs2022/mcs2022.pdf.

[7] Polish Cement Association. https://www.polskicement.pl/emisje/.

[8] Hanein T., Hayashi Y., Utton C., Nyberg M., Martinez J.C., Quintero-Mora N.I., Kinoshita H. (2020). Pyro processing cement kiln bypass dust: Enhancing clinker phase formation. Constr. Build. Mater..

[9] Zhang C.Y., Han R., Yu B., Wei Y.M. (2018). Accounting process-related CO_2_ emissions from global cement production under Shared Socioeconomic Pathways. J. Clean. Prod..

[10] Owsiak Z., Czapik P., Zapała-Sławeta J. (2020). Properties of a three-component mineral road binder for deep-cold recycling technology. Materials.

[11] Al-Jabri K.S., Taha R.A., Al-Hashmi A., Al-Harthy A.S. (2006). Effect of copper slag and cement by-pass dust addition on mechanical properties of concrete. Constr. Build. Mater..

[12] Al-Jabri K.S., Taha R.A., Al-Ghassani M. (2002). Use of copper slag and cement by-pass dust as cementitious materials. Cem. Concr. Aggreg..

[13] Adaska W.S., Taubert D.H. Beneficial uses of cement kiln dust. Proceedings of the 2008 IEEE Cement Industry Technical Conference Record.

[14] Sreekrishnavilasam A., Santagata M.C. (2006). Report No. FHWA/IN/JTRP-2005/10 Development of Criteria for the Utilization of Cement kiln Dust (CKD) in Highway Infrastructures.

[15] Czapik P., Zapała-Sławeta J., Owsiak Z., Stępień P. (2020). Hydration of cement by-pass dust. Constr. Build. Mater..

[16] 2012—INFORMATOR SPC—Przemysł Cementowy w liczbach”, Polish Cement Association Reports. https://www.polskicement.pl/2012-informator-spc-przemysl-cementowy-w-liczbach/.

[17] Abdel-Ghani N.T., El-Sayed H.A., El-Habak A.A. (2018). Utilization of by-pass cement kiln dust and air-cooled blast-furnace steel slag in the production of some “green” cement products. HBRC J..

[18] Darweesh H.H.M. (2017). A review article on the influence of the electrostatic precipitator cement kiln dust waste on the environment and public health. Am. J. Biol. Environ. Stat..

[19] Taha R., Al-Rawas A., Al-Harthy A., Qatan A. (2002). Use of cement bypass dust as filler in asphalt concrete mixture. J. Mater. Civil Eng..

[20] Khater G.A. (2006). Use of bypass cement dust for production of glass ceramic materials. Adv. Appl. Ceram..

[21] Uliasz-Bocheńczyk A. (2019). Chemical characteristics of dust from cement kilns. Gospod. Surowcami Miner..

[22] 2019—Informator SPC—Przemysł Cementowy w iczbach, Polish Cement Association Reports. https://www.polskicement.pl/2019-informator-spc-przemysl-cementowy-w-liczbach/.

[23] Yoobanpot N., Jamsawang P., Horpibulsuk S. (2017). Strength behavior and microstructural characteristics of soft clay stabilized with cement kiln dust and fly ash residue. Appl. Clay Sci..

[24] Peethamparan S., Olek J., Lovell J. (2008). Influence of chemical and physical characteristics of cement kiln dusts (CKDs) on their hydration behavior and potential suitability for soil stabilization. Cem. Concr. Res..

[25] Buczyński P., Iwański M. The influence of hydrated lime, portland cement and cement dust on rheological properties of recycled cold mixes with foamed bitumen. Proceedings of the “Environmental Engineering” 10th International Conference.

[26] Al-Aghbari M.Y., Mohamedzein Y.E.-A., Taha R. (2009). Stabilisation of desert sands using cement and cement dust. Proc. Inst. Civ. Eng. -Ground Improv..

[27] Sultan M.E., Abo-El-Enein S.A., Sayed A.Z., EL-Sokkary T.M., Hammad H.A. (2018). Incorporation of cement bypass flue dust in fly ash and blast furnace slag-based geopolymer. Case Stud. Constr. Mater..

[28] Heikal M., Zaki ME A., Ibrahim S.M. (2020). Preparation, physico-mechanical characteristics and durability of eco-alkali-activated binder from blast-furnace slag, cement kiln-by-pass dust and microsilica ternary system. Constr. Build. Mater..

[29] Nocuń-Wczelik W., Stolarska K. (2019). Calorimetry in the studies of by-pass cement kiln dust as an additive to the calcium aluminate cement. J. Therm. Anal. Calorim..

[30] Abdel-Gawwad H.A., Heikal M., Mohammed M.S., Abd El-Aleem S., Hassan H.S., García S.V., Alomayri T. (2019). Sustainable disposal of cement kiln dust in the production of cementitious materials. J. Clean. Prod..

[31] Wojtacha-Rychter K., Król M., Gołaszewska M., Całus-Moszko J., Magdziarczyk M., Smoliński A. (2022). Dust from chlorine bypass installation as cementitious materials replacement in concrete making. J. Build. Eng..

[32] Al-Harthy A.S., Taha R., Al-Maamary F. (2003). Effect of cement klin dust (CKD) on mortar and conrete mixtures. Constr. Build. Mater..

[33] Udoeyo F.F., Hyee A. (2002). Strengths of cement kiln dust concrete. J. Mater. Civ. Eng..

[34] Aydin T., Tarhan M., Tarhan B. (2019). Addition of cement kiln dust in ceramic wall tile bodies. J. Therm. Anal. Calorim..

[35] Mahrous M.A., Yang H.S. (2011). Utilization of cement kiln dust in industry cement bricks. Geosystem Eng..

[36] Abdulkareem A.H., Eyada S.O. (2018). Production of Building Bricks Using Cement Kiln Dust CKD Waste. Sustainable Civil Infrastructures.

[37] Abdel-Gawwad H.A., Rashad A.M., Mohammed M.S., Tawfik T.A. (2021). The potential application of cement kiln dust-red clay brick waste-silica fume composites as unfired building bricks with outstanding properties and high ability to CO_2_-capture. J. Build. Eng..

[38] Qu X., Zhao X. (2017). Previous and present investigations on the components, microstructure and main properties of autoclaved aerated concrete—A review. Constr. Build. Mater..

[39] Li J., Lv Y., Jiao X., Sun P., Li J., Wuri L., Zhang T.C. (2020). Electrolytic manganese residue based autoclaved bricks with Ca(OH)_2_ and thermal-mechanical activated K-feldspar additions. Constr. Build. Mater..

[40] Vojvodikova B., Prochazka L., Bohacova J. (2021). Posiibilities of application cement by-pass dust into the garden architecture elements. Crystals.

[41] Vojvodikova B., Prochazka L., Bohacova J. (2021). X-ray diffraction of alkali-activated materials with cement by-pass dust. Materials.

[42] Tkaczewska E. (2019). The influence of cement bypass dust on the properties of cement curing under normal and autoclave conditions. Struct. Environ..

[43] Temmermans F., Tromp O., Santamaria Razo D.A. (2020). A1 Method of Making Calcium Silicate Bricks. European Patent.

[44] Kurdowski W. (2014). Cement and Concrete Chemistry.

[45] (2015). Metody Badań Elementów Murowych- Część 1: Określenie Wytrzymałości na Ściskanie.

[46] (2011). 2011 Określanie Absorpcji Wody Ceramicznych i Silikatowych Elementów Murowych Przez Absorpcję Zimnej Wody.

[47] Stępień A., Leśniak M., Sitarz M.A. (2019). Sustainable autoclaved material made of glass sand. Buildings.

[48] Stępień A., Potrzeszcz-Sut B., Prentice P.D., Oey T., Balonis M. (2020). The role of glass compounds in autoclaved bricks. Buildings.

[49] Huang Z., Yuan Y., Chen Z., Wen Z. (2012). Microstructure of autoclaved aerated concrete hydration products in different water-to-binder ratio and different autoclaved system. Adv. Mater. Res..

